# On the Results of Lithrotity Methodically Applied to Suitable Cases

**Published:** 1847-04

**Authors:** 


*On the Results of Lithotrity, Methodically applied to Suitable Cases.* By M. Civiale. (Comptes Rendues, Nov. 23d, 1846. p. 979.)—From 1836 to 1845, I have employed my method two hundred and sixty-six times, and have obtained two hundred and fifty-nine cures, some of which were incomplete. This result is the more remarkable from the fact that most of the subjects were aged. There were only five children. Among those operated on, were five physicians or surgeons. Seventy-nine other calculous patients applied for relief, but I did not think them in a favorable condition for lithotrity. Twenty eight have submitted to the operation of lithotomy; seventeen of whom have been cured. The others retained their stones, and most of them afterwards fell victims either to the progress or complications of the disease. In comparing the new cases with those of which I have already rendered an account to the academy, it will be seen that the operation for lithotrity has been performed by me alone on five hundred and eighty-two subjects. It will be observed that the mortality is greater in the new than in the old tables. If figures alone are regarded, this result would imply a contradiction of what ought to be expected from the improvement attained, both in the instruments and manner of operating. The difference depends, howeuer, upon the fact, that in the commencement of my practice I would undertake only favorable cases. The fate of a new method was at stake, and all of the accidents which should justly have been attribated to the choice of improper subjects, would have been charged to it. Success alone could impose silence to an opposition every day more threatening, and, to obtain success, it was necessary to operate only when it was pretty certain. At this time the new method has been judged. Humanity requires the surgeon to resort to the method offering the greatest chances of saving the patient, and though the result is uncertain, lithotrity allows more hope of success than lithotomy. In operating on doubtful cases, the mortality must necessarily be greater. What lithotrity appears, however, to have lost in certainty, it has gained in extension. Formerly, not more than half of those presenting themselves were operated on; now three fourths undergo the process. Art, more confident of its powers, now attacks cases which prudence formerly commanded it to abandon.—*South. Jour. Med. and Pharm.*

